# Orthoplastic surgery – regenerative concepts and reconstructive strategies

**DOI:** 10.1515/iss-2025-0050

**Published:** 2025-11-04

**Authors:** Justus P. Beier, Frank Hildebrand

**Affiliations:** Klinik für Plastische Chirurgie, Hand- und Verbrennungschirurgie Uniklinik RWTH Achen, Pauwelsstraße 30 52074, Aachen, Germany; Klinik für Orthopädie, Unfall- und Wiederherstellungschirurgie, Uniklinik RWTH Aachen, Pauwelsstraße 30 52074, Aachen, Germany

Orthoplastic surgery represents a collaborative, interdisciplinary approach combining orthopedic and plastic surgery to maximize limb salvage and minimize the need for amputation. It addresses complex reconstructive challenges that require simultaneous application of the principles and techniques from both specialties. First articulated by Scott Levin over 30 years ago [1], the concept of orthoplastic surgery has since evolved and gained widespread acceptance worldwide. By definition, orthoplastic surgery involves “the principle and practice of both specialties applied to clinical problems simultaneously”, highlighting its inherently integrative nature.

Over the past decades, orthoplastic approaches have become standard in managing a variety of challenging clinical scenarios. These include chronic osteomyelitis, severe diabetic foot infections, critical limb ischemia in vasculopathic patients, mutilating injuries of the upper and lower extremities, sarcoma, and congenital limb differences. The success of limb salvage in these contexts has been profoundly influenced by advances in reconstructive (micro-)surgery, including autogenous free flap transplantation.

The development of orthoplastic surgery has been rooted in multiple surgical disciplines – orthopedic and trauma surgery, oncologic reconstruction, vascular surgery, plastic and hand surgery, and in particular microsurgery – and has required decades to integrate into routine practice. Its effectiveness depends on a deep understanding of both skeletal and soft tissue reconstruction, meticulous surgical planning, and seamless interdisciplinary collaboration.

Incorporating orthoplastic principles into medical education and training across specialties is crucial for further improving outcomes in limb salvage. In view of the ongoing worldwide armed conflicts resulting in a plethora of mutilating war injuries coming along with combined bone and soft tissue affection, even greater awareness among physicians and surgeons of orthoplastic techniques will become increasingly important to ensure that patients benefit from the most advanced reconstructive strategies for preserving limb function.

This special issue presents a comprehensive overview of orthoplastic surgery, covering current clinical practice for upper and lower limb reconstruction, management of vasculopathic patients, and emerging innovations such as robotic microsurgery. In addition, it highlights experimental research investigating molecular pathways in limb-threatening osteomyelitis and strategies for musculoskeletal tissue regeneration, reflecting the dynamic interface between clinical practice and experimental research.
